# Silicon-mediated defence response in chilli against yellow mite infestation

**DOI:** 10.1007/s10493-025-01079-7

**Published:** 2025-11-13

**Authors:** Mansura Afroz, Md Ruhul Amin, Md. Ramiz Uddin Miah, Md. Mamunur Rahman

**Affiliations:** 1 Department of Entomology, Gazipur Agricultural University, Gazipur, 1706 Bangladesh; 2 School of Natural and Environmental Sciences, Newcastle University, Newcastle upon Tyne, NE1 7RU United Kingdom

**Keywords:** Eco-friendly ‧ jasmonic acid ‧ leaf epidermis ‧ natural enemy ‧ salicylic acid

## Abstract

Chilli yellow mite is a major pest of chilli, which causes significant damage to both leaves and fruits. Synthetic acaricides, though widely used for control, pose environmental risks and often give unsatisfactory results due to pest resistance. Therefore, this study aimed to enhance the resistance of chilli plants against mite by applying silicon (Si), a natural element, in two forms- CaSiO₃ and K₂SiO₃, at concentrations of 0.1%, 0.5% and 1.0%. Results showed that Si application significantly reduced mite infestation while having no effect on their natural predator, the ladybird beetle. CaSiO₃ at 0.1% Si led to the lowest leaf infestation rates (7.6 ± 1.5%, 4.4 ± 0.4% and 2.4 ± 0.4% after the first, second and third sprays, respectively), and produced the highest number of healthy fruits at both the first and second harvests (345.8 ± 5.3 g/plant and 335.0 ± 4.2 g/plant, respectively). Si treatment increased the thickness of both the upper and lower leaf epidermis, especially with CaSiO₃ and K₂SiO₃ at 1.0% Si. It also elevated the levels of the stress-responsive hormones salicylic acid and jasmonic acid relative to the control, particularly with CaSiO₃ at 0.1% Si. Principal component analysis revealed that mite infestation was negatively correlated with both acid levels and leaf epidermis thickness. Thus, Si application presents a promising, eco-friendly strategy for managing mite infestations in chilli plants.

**Supplementary Information:**

The online version contains supplementary material available at 10.1007/s10493-025-01079-7.

## Introduction

Chilli (*Capsicum frutescens* L.) is a widely cultivated crop across the world. Beyond its broad medicinal benefits, chilli is a rich source of vitamin C, as well as vitamins A and B (Singh et al. 2007). Its distinctive pungent aroma and spicy flavor, primarily attributed to the compound capsaicin (Choudhary and Samadia 2004), not only make it an essential ingredient in curries but also maintain strong year-round demand. However, the high price of chilli is largely due to the significant risks and uncertainties the crop faces from both abiotic and biotic stresses. Biotic stresses include insect and mite pests, which adversely affect both quantity and quality of the fruit. Approximately 35 species of insect and mite pests have been recorded to infest chilli (Mokal et al. 2018), among which the chilli yellow mite, *Polyphagotarsonemus latus* (Banks) (Acari: Tarsonemidae), has been identified as a significant factor in reducing crop productivity (Gupta 2012). Both the nymph and adult stages of mite attack chilli shoots, flowers and developing fruits, resulting in significant yield losses (Nasrin et al. 2021). Characteristic damage symptoms caused by this pest include twisting and downward curling of leaf margins, as well as yellowing and bronzing of terminal leaves and shoots (Dhooria 2016).

Mites show rapid development and very high reproductive capacity under favorable conditions (Rashid et al. 2024). As a result, the application of acaricides often fails to produce satisfactory results in controlling the pest. Additionally, widespread use of pesticides is discouraged due to their environmental and health risks. The limitations of chemical pesticides have driven scientific efforts to find alternatives that enhance plant resistance to pests. In this context, the use of silicon (Si) to enhance plant resistance to biotic stresses, including insect and mite pests as well as plant diseases, has gained significant attention from researchers in recent years (Reynolds et al. 2016).

Si supplementation can influence inducible direct chemical defences in plants under arthropod infestation by activating signaling pathways that produce stress-responsive hormones, including salicylic acid (SA), jasmonic acid (JA) and ethylene (Aljbory and Chen 2018). Moreover, Si enhances the plant’s resistance to arthropod pests due to the accumulation of biosilica in the plant cell walls, which prevents pest feeding and decreases the digestibility of the leaves, as well as the biological performance of the pests (Keeping et al. 2014). Si supplementation can also alter the emission of herbivore-induced plant volatiles (HIPVs) to recruit more natural enemies (Liu et al. 2017). Some research findings suggest that applying silicon fertilizers effectively enhances resistance to various sucking pests in crops such as sugarcane, rice, cucumber, cabbage and soybean (Alyousuf et al. 2022; Yang et al. 2018). As a naturally occurring element, silicon poses little to no risk to the environment or beneficial arthropods, making it a suitable component of integrated pest management (IPM) programs.

There is a lack of research on controlling yellow mite infestations using Si, particularly in chilli crops. Therefore, it is worth investigating how Si application influences certain plant properties and modulates pest populations and their associated natural enemies in chilli. In light of these considerations, this project aimed to explore the infestation level of yellow mite on chilli plants under Si application, examine the physico-chemical changes occurring in chilli plants as a result of Si application and assess the Si-induced effects on the presence of natural enemies of the chilli mite. Researchers have investigated various sources of Si for pest management, including potassium silicate (Islam et al. 2022; Ribeiro et al. 2021), silicic acid (Alyousuf et al. 2022), nanosilica (Ribeiro et al. 2021), calcium silicate (de Queiroz et al. 2016) and rock dust (Faraone et al. 2020). However, potassium silicate is the most widely used form of Si for protecting plants against insect and mite infestations. This study employed two Si sources—calcium silicate and potassium silicate—at three concentrations to evaluate the effects of dose and source of Si on the response of chilli against yellow mite infestation.

## Materials and methods

### Experimental site and cultivation of chilli

The study was conducted between July 2022 and June 2023 in the research field and the laboratory of the Department of Entomology, Gazipur Agricultural University (GAU), Gazipur. The study site is located at 25°25′ N and 89°5′ E. It has an annual mean temperature ranging from a high of 36.0 °C to a low of 12.7 °C, with an average temperature of 25.8 °C, annual rainfall of 2376 mm and 66% relative humidity (Department of Agrometeorology Weather Station 2024). Among the 30 agro-ecological zones (AEZs) of Bangladesh, the area falls within the Madhupur Tract (AEZ-28), which is characterized by deep red to brown terrace soils with low pH (5.5–6.0) and low to very low availability of plant minerals and nutrients (Hossain et al. 2024). The cultivation area experiences regular mite infestations on Solanaceae crops, such as chilli, eggplant and tomato. A local chilli cultivar was used as the experimental material in this study. The seedlings were raised in polybags and then 25-day old seedlings were transplanted into the field.

### Silicon formulation and application

Since higher doses of Si have been shown to cause leaf burn (Long et al. 2018), lower concentrations were chosen for this study based on previous research reports (Silva et al. 2010; Zamojska et al. 2018). Therefore, the treatments included foliar applications of Si at 0.1%, 0.5% and 1.0%. Each Si formulation was separately prepared by mixing the required amount of deionized water with calcium silicate (CaSiO₃: 34.9% CaO and 22.4% SiO₂) and potassium silicate (K₂SiO₃: 21.0% K₂O and 32.0% SiO₂). Untreated plants served as the control group, receiving no Si. The experiment was laid out in a randomized complete block design with three blocks. There were a total of 21 plots, each measuring 1.0 m × 1.5 m. Each plot contained six plants arranged in three rows, with a spacing of 0.5 m between plants and between rows. Each treatment had three replications, and two plants from each replication were randomly selected and tagged for observation and data collection. For foliar spraying, 50 mL of treatment solutions at each concentration was applied from the base to the top of the chilli plants. The treatments were initiated 15 days after transplanting and applied three times at 10-day intervals. Irrigation and other standard cultural practices were followed for both treated and control plants according to general crop cultivation protocols.

### Observation of plant infestation

Observations were made 72 h after each Si application. Data were collected from the selected plants. The total number of leaves and the number of infested leaves (either curled or both curled and yellow) from three randomly selected branches per plant were counted and converted into a percentage of infestation. Infested leaves were collected in small plastic bags, brought to the laboratory and examined under a compound microscope, which confirmed the mite species as *P. latus*.

### Observation of natural enemies

To count natural enemies, a thorough visual inspection was performed on the entire two selected plants for each replication 72 h after each spray. Only ladybird beetles, the natural enemies of mite, were found, and their numbers (both larvae and adults) were recorded on a per-plant basis.

### Leaf anatomy analysis

Two leaves from each of the selected plants were randomly selected for leaf anatomy analysis 72 h after the third Si spray. Small transverse sections of the chilli leaves were prepared, stained with Safranin-O and observed under a compound microscope equipped with a high-resolution camera and computer (Supplementary Fig. 1. A-G). The thickness of both the upper and lower epidermal layers was measured at three different points and averaged. Observation and measurement were done using Zen Lite 2012 image acquisition and analysis software.

### Determination of plant stress-responsive hormones

The amounts of SA and JA in fresh leaf samples from all treatments, including untreated control plants, were estimated through Gas Chromatography Mass Spectrometry (GC-MS) following the protocol described by Huang et al. (2015). In brief, leaves were collected from each of the selected plants 72 h after the third spray, placed in liquid nitrogen and transferred to a −20 °C freezer for storage until analysis. Salicylic and jasmonic acid standards were HPLC grade and purchased from Sigma-Aldrich, Germany. Standard stock solutions and working solutions were prepared by dissolving the individual standards in methanol. Leaf extraction and processing were carried out on 1.0 g of leaf material using methanol and ethyl acetate solvents. The internal standard, dihydro jasmonate (10 µL), was added to each sample. The extracts were analyzed using a SHIMADZU GC-MS QP-2020 system utilizing an SH Rxi 5MS chromatographic column (30 m × 0.25 mm × 0.25 μm). The injection temperature and volume were set at 280 °C and 2 µL, respectively. Helium, as the carrier gas, was used at a flow rate of 1.1 mL/min. The temperature program commenced with an initial column temperature of 70 °C for 4 min, followed by a ramp to 300 °C at a rate of 10 °C/min, maintained for 2 min. The temperature was then increased to 340 °C at a rate of 5 °C/min and held until the end of the analysis. Selected-ion monitoring mode was employed with 70 eV electron ionization and a dwell time of 0.3 s for quantification. The source and quadrupole temperatures were maintained at 230 °C and 150 °C, respectively. Mass data were collected across a range of 40 to 300 m/z. Salicylic acid, jasmonic acid and dihydrojasmonate exhibited retention times of 12.1, 17.0 and 13.2 min, respectively, with corresponding quantifier ions of 120, 83 and 151 m/z. The concentrations of SA and JA were expressed in µg/mL. The tests were conducted at the Institute of Food Science and Technology Laboratory of the Bangladesh Council of Scientific and Industrial Research (BCSIR).

### Fruit harvesting

Fruits were harvested twice. After each harvest, the plucked fruits from each treatment were separated into marketable and infested categories, and their weights were measured.

### Data analysis

The data were analyzed using IBM SPSS Statistics (version 29) and R software (version 4.4.1). Shapiro-Wilk test was used to evaluate the normality of data distribution and Levene’s test was conducted to assess homogeneity of variances. Analysis of variance (ANOVA), followed by Tukey’s HSD post-hoc test (at a 5% level of significance), was performed to compare differences among treatments in terms of percent leaf infestation by mite, natural enemy abundance, concentrations of SA and JA in leaf samples, thickness of leaf epidermis layers and yield. The percent reduction in leaf infestation after each spray was calculated relative to the mean percent leaf infestation in the corresponding control. The following formula was applied to the infestation data of each replication.

1 $$\eqalign{ \:\% \:{\text{Reduction}}\:{\text{of}}\:{\text{leaf}}\:{\text{infestation}} {\text{ = }} \cr &\frac{{{\text{Mean}}\:{\text{percent}}\:{\text{leaf}}\:{\text{infestation}}\:{\text{in}}\:{\text{control - Percent}}\:{\text{leaf}}\:{\text{infestation}}\:{\text{in}}\:{\text{treatment}}}}{{{\text{Mean}}\:{\text{percent}}\:{\text{leaf}}\:{\text{infestation}}\:{\text{in}}\:{\text{control}}}}\: \cr & \times \:{\text{100}} \cr}$$

A cluster heatmap and Principal Component Analysis (PCA) were employed to visualize treatment effects in a multivariate context, identify clustering patterns among treatments and explore correlations among the measured variables. Additionally, a Multivariate Analysis of Variance (MANOVA) was conducted to evaluate the overall effect of treatments on the set of dependent variables, using Pillai’s Trace as the test statistic. Statistical significance was determined at the *p* < 0.05 level. An independent sample t-test was performed to compare the effect of the same concentration of Si from the CaSiO₃ and K₂SiO₃ sources on leaf infestation by mite.

## Results

### Effect of silicon on leaf infestation by *P. latus* in chilli

**Fig. 1:**
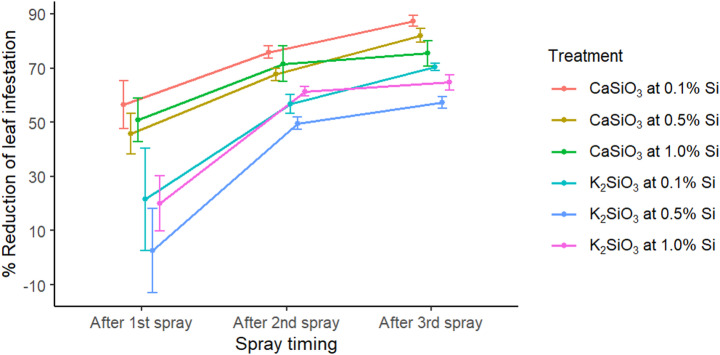
Mean (± SE) percentage reduction in *P. latus* leaf infestation compared with the control from the first to third spray

Chilli leaf infestation by *P. latus* was assessed 72 h after each Si spray, using six replications per treatment. Application of Si, in the form of CaSiO₃ or K₂SiO₃, significantly reduced *P. latus* infestation (Table 1). Prior to treatment, there was no significant difference in percent leaf infestation among sampled plants (F_6,35_ = 2.043, *p* = 0.0858). Following application, all treated plants showed a marked reduction in mite infestation compared to pre-spray levels, with CaSiO₃ containing 0.1% Si resulting in the significantly lower infestation than the other Si treatments. This treatment lowered the infestation to 7.6 ± 1.5% after the 1 st spray, further reducing it to 4.4 ± 0.4% and 2.4 ± 0.4% after the 2nd and 3rd sprays, respectively. In contrast, the infestation in the untreated control plants increased to 19.2 ± 0.6%, from an initial 14.1 ± 2.6%. The mean reduction in percent leaf infestation of Si-treated plants was calculated relative to the control after each spray, and results varied across treatments (Fig. 1). The mean percent reduction ranged from 2.6% to 56.5% after the first spray, 49.6% to 75.8% after the second and 57.2% to 87.3% after the third. CaSiO₃ containing 0.1% Si showed the greatest reduction, with mean values of 56.5 ± 8.78%, 75.8 ± 2.28%, and 87.3 ± 2.03% after the first, second and third sprays, respectively.

**Table 1:** The leaf infestation by *P. latus* in chilli plants after silicon application

Treatment	Pre spray (%)	After 1 st spray (%)	After 2nd spray (%)	After 3rd spray (%)
CaSiO₃ at 0.1% Si	14.8 ± 4.1 a	7.6 ± 1.5 b	4.4 ± 0.4 c	2.4 ± 0.4 e
CaSiO₃ at 0.5% Si	17.6 ± 2.4 a	9.5 ± 1.3 ab	5.9 ± 0.4 c	3.5 ± 0.5 de
CaSiO₃ at 1.0% Si	22.6 ± 2.0 a	8.6 ± 1.4 ab	5.2 ± 1.2 bc	4.7 ± 0.9 cde
K₂SiO₃ at 0.1% Si	14.2 ± 2.9 a	13.7 ± 3.3 ab	7.9 ± 0.6 bc	5.7 ± 0.3 cd
K₂SiO₃ at 0.5% Si	21.9 ± 3.3 a	17.0 ± 2.7 ab	9.2 ± 0.4 b	8.2 ± 0.4 b
K₂SiO₃ at 1.0% Si	11.8 ± 2.6 a	14.0 ± 1.8 ab	7.0 ± 0.3 bc	6.8 ± 0.5 bc
Control	14.1 ± 2.6 a	17.5 ± 2.1 a	18.2 ± 1.7 a	19.2 ± 0.6 a

Data is represented as Mean ± SE. Means within a column followed by same letter(s) are not significantly different according to Tukey′s HSD post hoc test (*p* < 0.05). Pre spray: F_6,35_ = 2.043, *p* = 0.0858, Shapiro-Wilk test *p* > 0.05, Levene’s test *p* > 0.05; 1 st spray: F_6,35_ = 3.538, *p* < 0.01, Shapiro-Wilk test *p* > 0.05, Levene’s test *p* > 0.05; 2nd spray: F_6,35_ = 29.6, *p* < 0.001, Shapiro-Wilk test *p* = 0.0188, Levene’s test *p* > 0.05; 3rd spray: F_6,35_ = 107, *p* < 0.001, Shapiro-Wilk test *p* > 0.05, Levene’s test *p* > 0.05

### Effect of silicon on ladybird beetle population

The application of Si significantly reduced the *P. latus* infestation levels in chilli plants. However, the Si application did not significantly affect the abundance of ladybird beetles, a natural predator of mites (Supplementary Table 1) and their abundance remained statistically similar between the treated and untreated control plants. Additionally, there was little variation in ladybird beetle abundance between the pre-spray and post-spray periods. On average, 1.2 ± 0.5 to 2.5 ± 0.4 ladybird beetles per plant were observed throughout the study, regardless of treatment.

### Plant characteristics and yield following silicon application

**Fig. 2:**
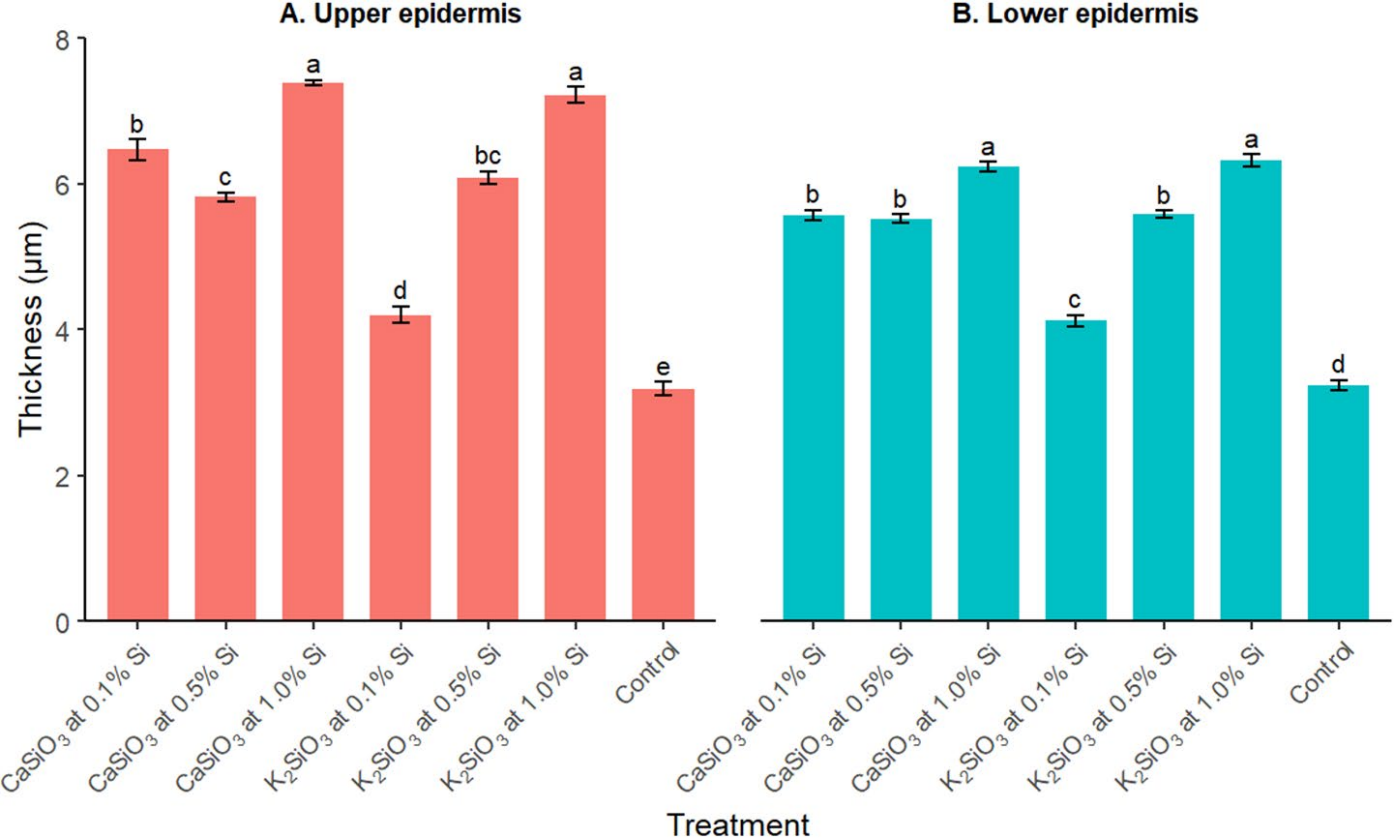
The thickness of the upper and lower epidermis of chilli leaf (Mean ± SE) after the application of CaSiO₃ and K₂SiO₃ having silicon at different concentrations. Means of the same-colored bar followed by same letter(s) are not significantly different according to Tukey′s HSD post hoc test (*p* < 0.05). Upper epidermis: F_6,35_ = 255.0, *p* < 0.001, Shapiro-Wilk test *p* > 0.05, Levene’s test *p* > 0.05; Lower epidermis: F_6,35_ = 262.0, *p* < 0.001, Shapiro-Wilk test *p* > 0.05, Levene’s test *p* > 0.05

Transverse sections of chilli leaves were examined under a microscope, and images were analyzed. The image analysis results revealed a significant increase in leaf epidermis thickness due to Si application (Fig. 2). At 1.0% Si, both CaSiO₃ and K₂SiO₃ resulted in the highest upper epidermis thickness (7.4 ± 0.03 μm and 7.2 ± 0.1 μm, respectively). In contrast, the untreated control leaves exhibited the thinnest upper epidermis (3.2 ± 0.1 μm). A similar trend was observed in the lower epidermis, where CaSiO₃ and K₂SiO₃ at 1.0% Si produced the highest leaf thickness (6.2 ± 0.01 μm and 6.3 ± 0.1 μm, respectively). Similarly to the upper epidermis, the untreated control showed the lowest lower epidermis thickness (3.2 ± 0.1 μm).

**Fig. 3:**
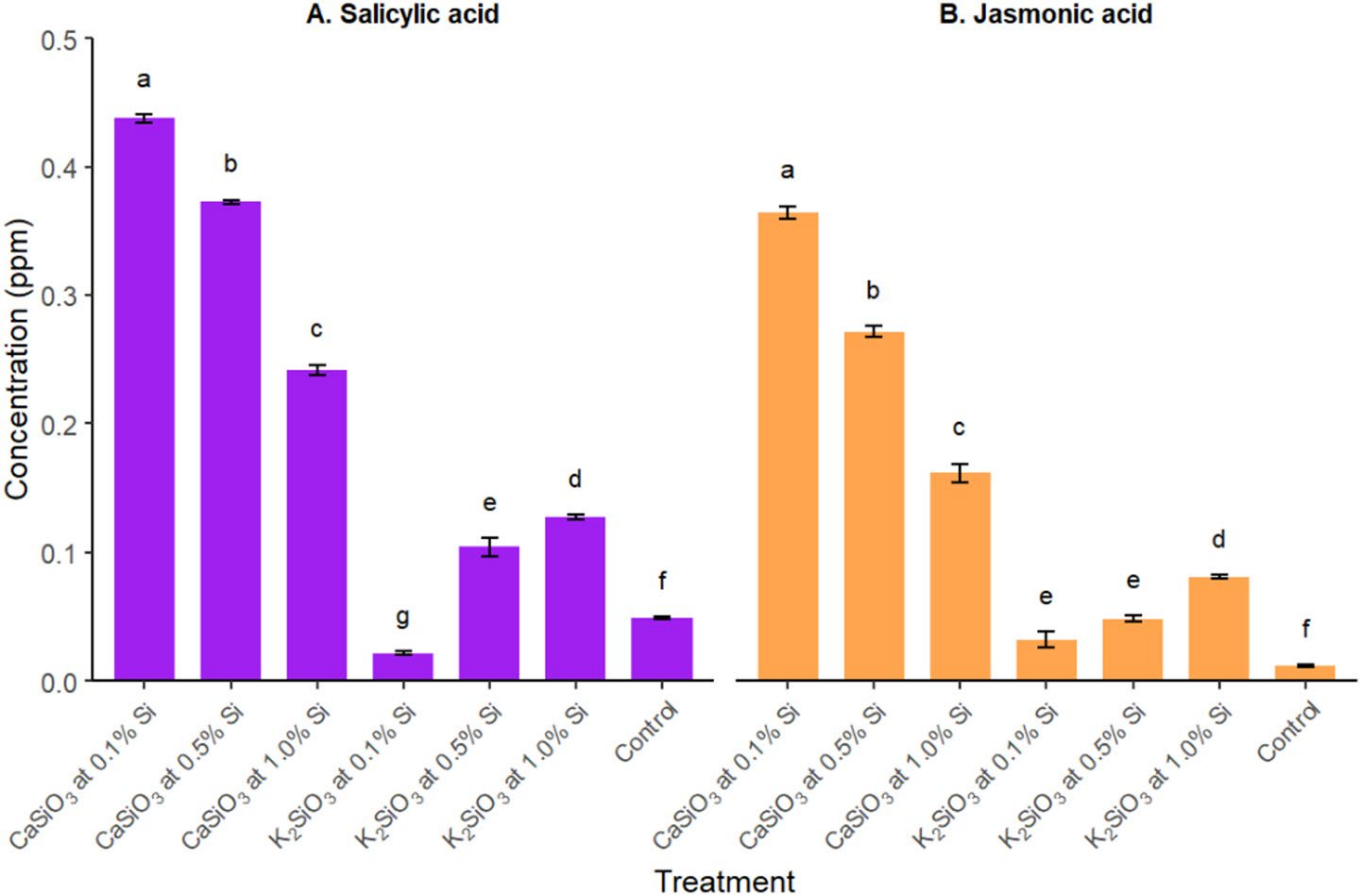
The amount of salicylic acid and jasmonic acid (Mean ± SE) found in chilli leaf after the silicon application. Means of the same-colored bar followed by same letter(s) are not significantly different according to Tukey′s HSD post hoc test (*p* < 0.05). Salicylic acid: F_6,35_ = 2122.0, *p* < 0.001, Shapiro-Wilk test *p* > 0.05, Levene’s test *p* > 0.05; Jasmonic acid: F_6,35_ = 914.3, *p* < 0.001, Shapiro-Wilk test *p* > 0.05, Levene’s test *p* > 0.05

The application of Si also significantly affected the amounts of plant stress-responsive hormones, SA and JA (Fig. 3). GC-MS data indicated that the application of Si, both as CaSiO₃ and K₂SiO₃ at different concentrations, generally increased the concentration of SA, except in the case of K₂SiO₃ containing 0.1% Si, where the amount of salicylic acid slightly decreased (0.0215 ± 0.00154 µg/mL) compared to the untreated control (0.049 ± 0.00058 µg/mL). The findings showed that the levels of both acids decreased with increasing concentrations of Si in CaSiO₃ treatments, while they increased with higher Si concentrations in K₂SiO₃ applications. However, the amount of both SA and JA in the leaves was significantly higher under CaSiO₃ treatments compared to K₂SiO₃ applications. Additionally, CaSiO₃ containing 0.1% Si induced the highest production of both salicylic acid and jasmonic acid (0.4375 ± 0.003 µg/mL and 0.3643 ± 0.00506 µg/mL, respectively).

The mature chillies were harvested twice during the study. In both cases, Si-treated plants produced a higher quantity of healthy fruits compared to untreated control plants. Among the Si treatments, plants treated with CaSiO₃ containing 0.1% Si showed the highest number of healthy fruits (345.8 ± 5.3 g per plant and 335.0 ± 4.2 g per plant at the first and second harvests, respectively), which was comparable to the yields from plants treated with 1.0% Si from both CaSiO₃ and K₂SiO₃ (Table 2). In contrast, the amount of infested fruit was highest in the untreated control plants, with 63.0 ± 1.3 g per plant at the first harvest and 31.5 ± 0.8 g per plant at the second harvest.

**Table 2:** The number of healthy and infested chilli fruits at harvest after silicon application

Treatment	First harvest		Second harvest	
	Healthy fruit weight/plant (g)	Infested fruit weight/plant (g)	Healthy fruit weight/plant (g)	Infested fruit weight/plant (g)
CaSiO₃ at 0.1% Si	345.8 ± 5.3 a	56.8 ± 0.6 b	335.0 ± 4.2 a	27.5 ± 3.8 ab
CaSiO₃ at 0.5% Si	337.2 ± 7.2 ab	56.5 ± 0.8 b	329.2 ± 2.2 ab	24.2 ± 0.5 b
CaSiO₃ at 1.0% Si	344.0 ± 4.6 a	56.5 ± 0.7 b	334.7 ± 2.9 a	23.7 ± 1.0 b
K₂SiO₃ at 0.1% Si	337.7 ± 4.8 ab	55.7 ± 0.7 b	327.2 ± 2.6 ab	23.3 ± 0.7 b
K₂SiO₃ at 0.5% Si	336.2 ± 5.3 ab	57.2 ± 0.5 b	326.3 ± 1.2 ab	23.3 ± 0.4 b
K₂SiO₃ at 1.0% Si	339.7 ± 8.6 ab	57.0 ± 0.8 b	332.2 ± 3.6 a	23.2 ± 0.7 b
Control	316.3 ± 3.5 b	63.0 ± 1.3 a	317.7 ± 2.5 b	31.5 ± 0.8 a

Data is represented as Mean ± SE. Means within a column followed by same letter(s) are not significantly different according to Tukey′s HSD post hoc test (*p* < 0.05). 1 st harvest healthy fruit: F_6,35_ = 2.752, *p* < 0.05, Shapiro-Wilk test *p* > 0.05, Levene’s test *p* > 0.05; 1 st harvest infested fruit: F_6,35_ = 9.511, *p* < 0.001, Shapiro-Wilk test *p* > 0.05, Levene’s test *p* > 0.05; 2nd harvest healthy fruit: F_6,35_ = 4.348, *p* < 0.01, Shapiro-Wilk test *p* > 0.05, Levene’s test *p* > 0.05; 2nd harvest infested fruit: F_6,35_ = 4.015, *p* < 0.001, Shapiro-Wilk test *p* > 0.05, Levene’s test *p* > 0.05

### Multivariate analyses of Si treatments

Multivariate analyses were conducted to assess the effect of treatments on eight dependent variables. These included leaf infestation and ladybird beetle abundance observed after the third spray, the average number of healthy and infested fruits from two harvests, upper and lower leaf epidermis thickness, and levels of salicylic and jasmonic acids.

**Fig. 4:**
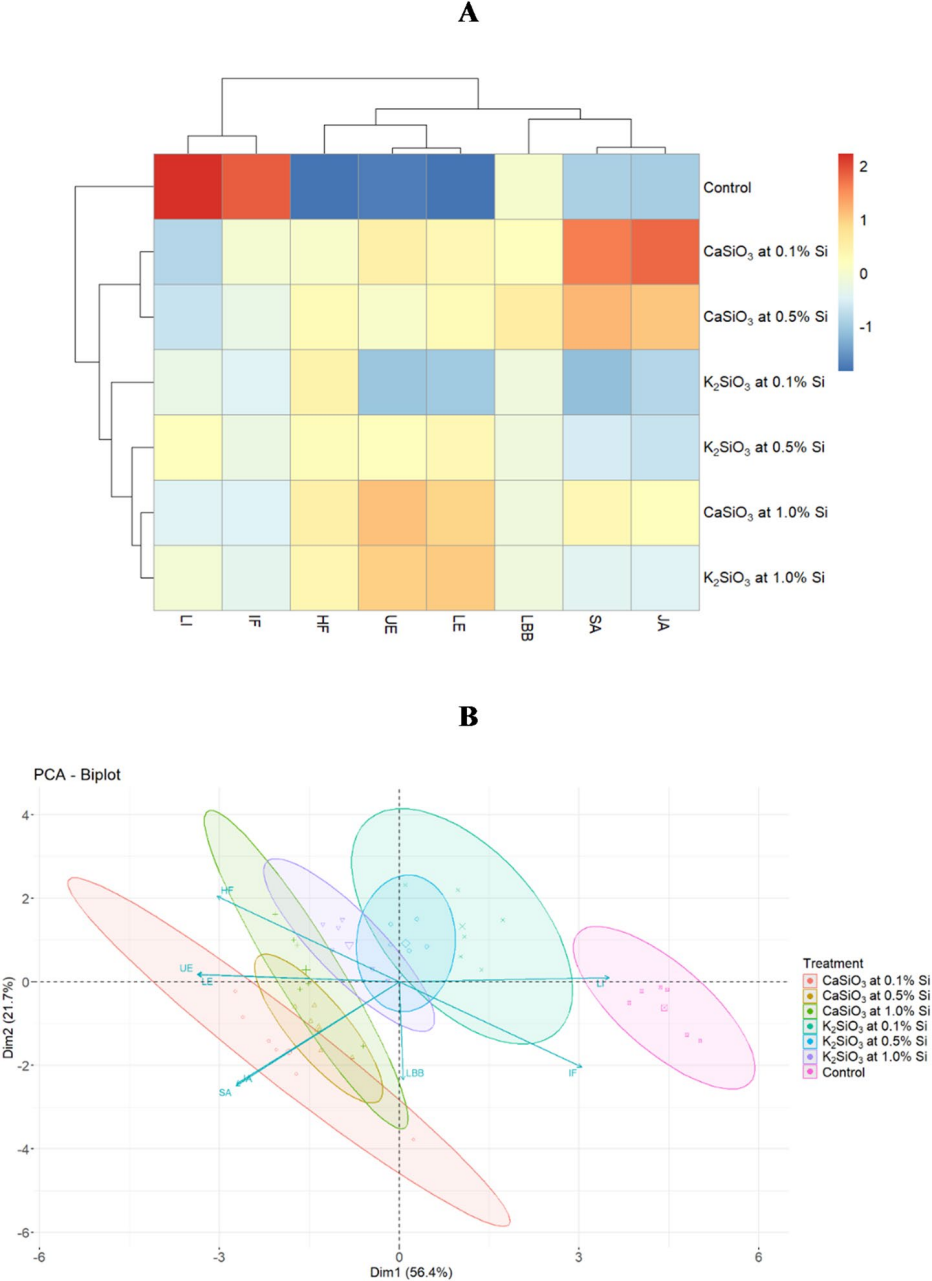
Relationship of the treatments with the studied variables. **(A)** Cluster heatmap of treatments and dependent variables. Dependent variables are plotted as columns, and treatments are plotted as rows. The heatmap is clustered by both rows and columns. Each cell represents the value of the data, with red indicating higher values and blue indicating lower values. **(B)** PCA biplot representing two main principal components for the dependent variables observed under Si treatments and control. The direction and length of the vectors indicate the contribution of the dependent variables to the first two components in the PCA. Here, LI = Percent leaf infestation after 3rd spray, IF = Percent total infested fruit, HF = Percent total healthy fruit, UE = Thickness of upper epidermis of leaf, LE = Thickness of lower epidermis of leaf, LBB = ladybird beetle abundance after 3rd spray, JA = Jasmonic acid, SA = Salicylic acid

The cluster heatmap indicates that Si application showed a distinctly different response compared to that of the untreated control (Fig. 4A). CaSiO₃ at 0.1% and 0.5% Si exhibited similar response patterns, marked by higher SA and JA concentrations. CaSiO₃ at 1.0% Si and K₂SiO₃ at 1.0% Si were related based on increased thickness of leaf epidermis layers. On the other hand, K₂SiO₃ at 0.1% and 0.5% Si showed lower levels of SA and JA and less thickness of leaf epidermis than the other Si treatments. The control group was clearly distinguished by its higher levels of leaf and fruit infestation.

PCA further supported the cluster heatmap results, depicting a distinct response in the control group, while the other treatment groups showed considerable overlap (Fig. 4B). Principal Component 1 (PC1 or Dim1) and Principal Component 2 (PC2 or Dim2) together accounted for 78.1% (56.4% and 21.7%, respectively) of the variability. The proportion of variation explained by other dimensions is shown in Supplementary Fig. 2. Dim1 showed a positive correlation with leaf infestation but negative correlations with leaf epidermis thickness and the levels of SA and JA. This suggests that leaf infestation decreased as epidermis thickness and concentrations of SA and JA increased. A positive correlation was observed between SA and JA, whereas a negative correlation was found between the percentages of healthy and infested fruits, as expected.

The MANOVA revealed that the treatments had a statistically significant (Pillai’s Trace = 3.8697, F_48,198_ = 7.4929, *p* < 0.001) overall effect on the combined set of dependent variables, which included percent leaf infestation, ladybird beetle population, upper and lower leaf epidermis thickness, salicylic and jasmonic acid concentrations and the percentages of healthy and infested fruit. This indicates that the treatments influenced multiple aspects of plant and pest responses simultaneously.

Furthermore, the leaf infestation following three applications of Si in the forms of CaSiO₃ and K₂SiO₃ was compared. The results indicated that applying Si at the same concentration from both CaSiO₃ and K₂SiO₃ yielded statistically similar outcomes (Supplementary Fig. 3). This suggests that similar concentrations of Si, regardless of whether from CaSiO₃ or K₂SiO₃, had a similar effect on *P. latus* infestation.

## Discussion

Several previous studies have demonstrated that Si application provides various benefits to plants, enhancing their resistance to a range of insect herbivores, including both chewing and sap-sucking pests. For instance, the foliar application of a 1% silicic acid solution significantly reduced the number of *Bemisia tabaci* (whitefly) eggs and nymphs on chrysanthemum plants (Melo et al. 2015). Similarly, Nikpay and Laane (2020) used silicic acid foliar sprays to control the sugarcane mite and observed a decrease in mite populations, along with a reduction in the percentage of dry leaves in treated sugarcane compared to controls. Moreover, Si treatments, administered either through foliar spraying or soil drenching, substantially reduced populations of whiteflies and leaf miner larvae on tomato leaves under greenhouse conditions (Alyousuf et al. 2022). In the present study, Si was applied to control the infestation of chilli yellow mites, a sap-sucking pest. Results revealed that Si-treated plants exhibited a lower mite infestation than the untreated control plants.

One of the key defence mechanisms associated with Si application is the formation of a physical barrier in plant tissues (Hall et al. 2020). Si forms biosilica, which accumulates in veins and leaf epidermal cells (Keeping et al. 2014). This stiffening of the cell wall reduces the palatability and digestibility of plants (Massey and Hartley 2009; Moraes et al. 2005), thereby decreasing host acceptance and suitability for insects (Massey et al. 2006; Keeping et al. 2014). In the current study, the thickening of the leaf epidermis layers compared to the control indicated that Si contributed to increased leaf stiffness and resistance to mite attack. Similar results were also reported by Asmar et al. (2015), who found that silicates increased leaf epidermis thickness, improving the acclimatization of banana plants in in vitro culture. We observed that the increased dose of Si corresponded to higher increase of the epidermis thickness in the present study. Si has also been shown to affect insect biology. The application of potassium silicate, for example, reduced the fecundity of *Diaphorina citri* on lime (Ramírez-Godoy et al. 2018) and *Tetranychus urticae* on papaya (Catalani et al. 2017) and strawberry (Ribeiro et al. 2021).

Si is known to stimulate the production of phenolic compounds, which are essential components of plant defence mechanisms (Frew et al. 2016; Reynolds et al. 2016). These defence chemicals help suppress pest abundance and subsequent infestation by influencing oviposition preferences, extending developmental periods and increasing mortality rates of pest insect (Correa et al. 2005; Reynolds et al. 2009). Si also influences the production of systemic stress signals, such as SA and JA, both of which play crucial roles in plant defence against biotic stresses (Aljbory and Chen 2018; Waterman et al. 2019). Stressed plants may utilize Si for structural support and growth at a lower metabolic cost (Cooke and Leishman 2011), freeing up resources for defence mechanisms, such as HIPV production. Si is thought to prevent pest-induced suppression of jasmonic and salicylic acid pathways, allowing for continuous HIPV production, which attracts natural enemies (Ponzio et al. 2013). Si-treated plants under herbivory have been shown to attract a greater proportion of predatory mites (64%) compared to untreated plants, indicating that Si may alter the composition of HIPVs in response to *T. urticae* infestation, enhancing predator attraction (Islam et al. 2022). In another study, Si did not show adverse effects on the biological control agent *Stethorus gilvifrons*, as statistically similar populations were found on both treated and untreated plants (Nikpay and Laane 2020). In the present study, Si application neither increased the number of natural enemies nor adversely affected their populations, supporting the claim that Si might not have a negative effect on beneficial organisms.

In this study, Si-treated plants showed increased concentrations of SA and JA. Interestingly, CaSiO₃ was more effective than K₂SiO₃ in stimulating the production of these plant stress-responsive hormones. Our findings also showed that CaSiO₃ and K₂SiO₃, at similar doses, produced statistically similar results in terms of leaf infestation by *P. latus*. Overall, the current results indicated that lower concentrations of Si (0.1–0.5%) were more effective in controlling mite infestations compared to the 1.0% concentration. Nikpay and Laane (2020) demonstrated that the effectiveness of Si treatments might increase with application frequency. In this study, Si was applied during the early growth stage, resulting in moderate to low levels of infestation. However, based on the findings from Nikpay and Laane (2020), increasing the frequency of Si applications may yield better results during severe mite infestations. However, further research is needed to determine the optimal dose and frequency of Si application.

The present study demonstrated that Si enhanced chilli plant defences against yellow mite infestations. Si can be used in combination with other pest control strategies to achieve an additive effect, as observed by Gatarayiha et al. (2010), who reported that combining the entomopathogenic fungus *Beauveria bassiana* with potassium silicate effectively reduced *T. urticae* populations on beans, cucumbers, maize and eggplants. Si is a naturally occurring, inexpensive and non-toxic substance with no reported harmful effects on humans or non-target organisms. Moreover, Si has been shown to enhance plant growth and biomass production (Verma et al. 2020), offering an added benefit that further supports its role in sustainable pest management as an alternative to synthetic pesticides. Therefore, Si can be effectively included into Integrated Pest Management (IPM) strategies, as its efficacy, environmental friendliness and safety align well with the principles of IPM.

To conclude, the application of Si increased the thickness of both the upper and lower epidermis, as well as the levels of the plant defence chemicals, SA and JA. Consequently, Si-treated plants, regardless of the source of Si, exhibited significantly lower *P. latus* infestation compared to untreated control plants. However, Si did not have any significant effect on the abundance of ladybird beetles. Although CaSiO₃ at 0.1% was more effective in promoting the production of plant stress-responsive hormones, no significant differences were observed between CaSiO₃ and K₂SiO₃ in their effects on *P. latus* infestation at the same Si concentrations. Therefore, the use of Si, in the form of either CaSiO₃ or K₂SiO₃, can be recommended as an eco-friendly strategy for the effective management of chilli yellow mites.

## Supplementary Information

Below is the link to the electronic supplementary material.

Supplementary material 1 (DOCX 1825.6 kb)

## Data Availability

We declare that all data is provided within the manuscript.
