# Supplementary material for: Silicon-mediated defence response in chilli against yellow mite infestation
Source: Exp Appl Acarol. 2025 Nov 13;95(4):56. doi: 10.1007/s10493-025-01079-7 (PMC12615510; doi:10.1007/s10493-025-01079-7)
Supplement: Supplementary file 1 — Supplementary material 1 (DOCX 1825.6 kb) [file 10493_2025_1079_MOESM1_ESM.docx]

**Supplementary Table 1**

The abundance of the natural enemy of mite (ladybird beetle) after the application of silicon

| Treatment | No. per plant | | | |
| --- | --- | --- | --- | --- |
|  | Pre-spray | After 1st spray | After 2nd spray | After 3rd spray |
| CaSiO₃ at 0.1% Si | 2.3 ± 0.6 a | 1.8 ± 0.5 a | 1.7 ± 0.4 a | 1.7 ± 0.4 a |
| CaSiO₃ at 0.5% Si | 2.8 ± 0.3 a | 1.7 ± 0.5 a | 1.5 ± 0.4 a | 2.0 ± 0.3 a |
| CaSiO₃ at 1.0% Si | 2.0 ± 0.6 a | 2.5 ± 0.6 a | 1.5 ± 0.4 a | 1.3 ± 0.5 a |
| K₂SiO₃ at 0.1% Si | 2.0 ± 0.6 a | 1.8 ± 0.5 a | 1.2 ± 0.5 a | 1.3 ± 0.5 a |
| K₂SiO₃ at 0.5% Si | 1.8 ± 0.6 a | 2.0 ± 0.6 a | 1.7 ± 0.5 a | 1.3 ± 0.4 a |
| K₂SiO₃ at 1.0% Si | 1.8 ± 0.5 a | 1.5 ± 0.4 a | 1.8 ± 0.7 a | 1.3 ± 0.3 a |
| Control | 1.8 ± 0.3 a | 2.5 ± 0.4 a | 1.8 ± 0.3 a | 1.5 ± 0.2 a |

Data is represented as Mean ± SE. Means within a column followed by same letter(s) are not significantly different according to Tukey′s HSD post hoc test (p < 0.05). Pre spray: F_6,35_ = 0.549, p = 0.768, Shapiro-Wilk test p > 0.05, Levene’s test p > 0.05; 1st spray: F_6,35_ = 0.62, p = 0.713, Shapiro-Wilk test p > 0.05, Levene’s test p > 0.05; 2nd spray: F_6,35_ = 0.247, p = 0.957, Shapiro-Wilk test p > 0.05, Levene’s test p > 0.05; 3rd spray: F_6,35_ = 0.423, p = 0.859, Shapiro-Wilk test p > 0.05, Levene’s test p > 0.05.


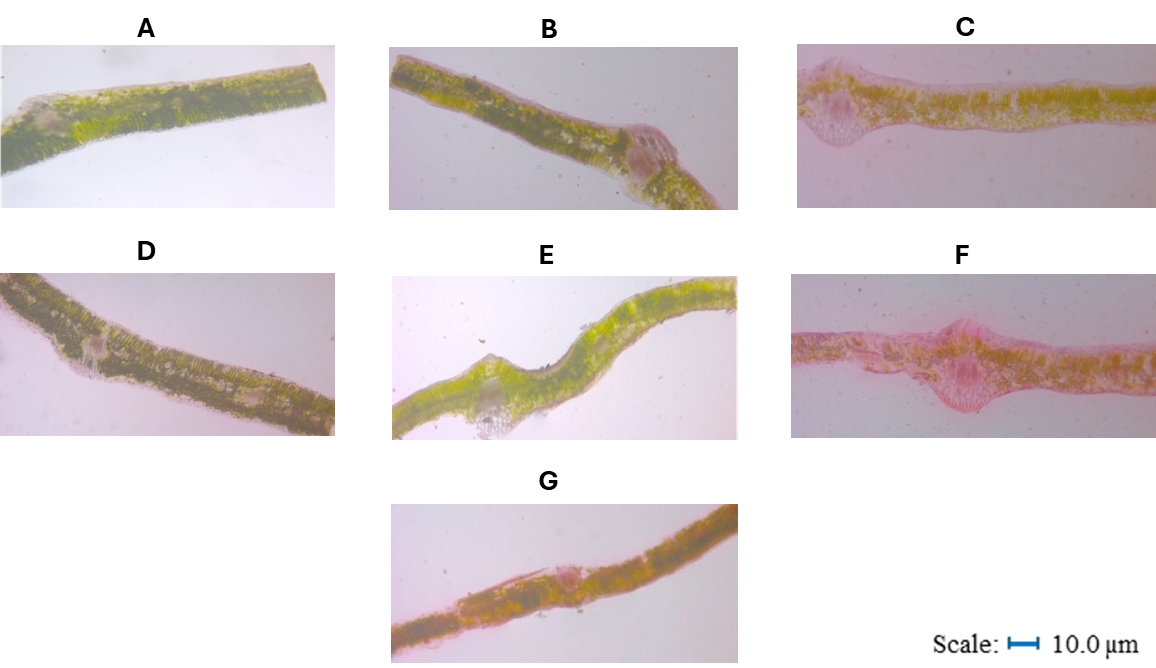


**Supplementary Fig. 1** Observation of epidermis thickness of chilli leaf under microscope. **A.** Ca_2_SiO_4_ at 0.1% Si treated leaf, B**.** Ca_2_SiO_4_ at 0.5% Si treated leaf, **C.** Ca_2_SiO_4_ at 1.0% Si treated leaf, **D.** K_2_SiO_3_ at 0.1% Si treated leaf, **E.** K_2_SiO_3_ at 0.5% Si treated leaf, **F.** K_2_SiO_3_ at 1.0% Si treated leaf and **G.** Chilli leaf under untreated control.


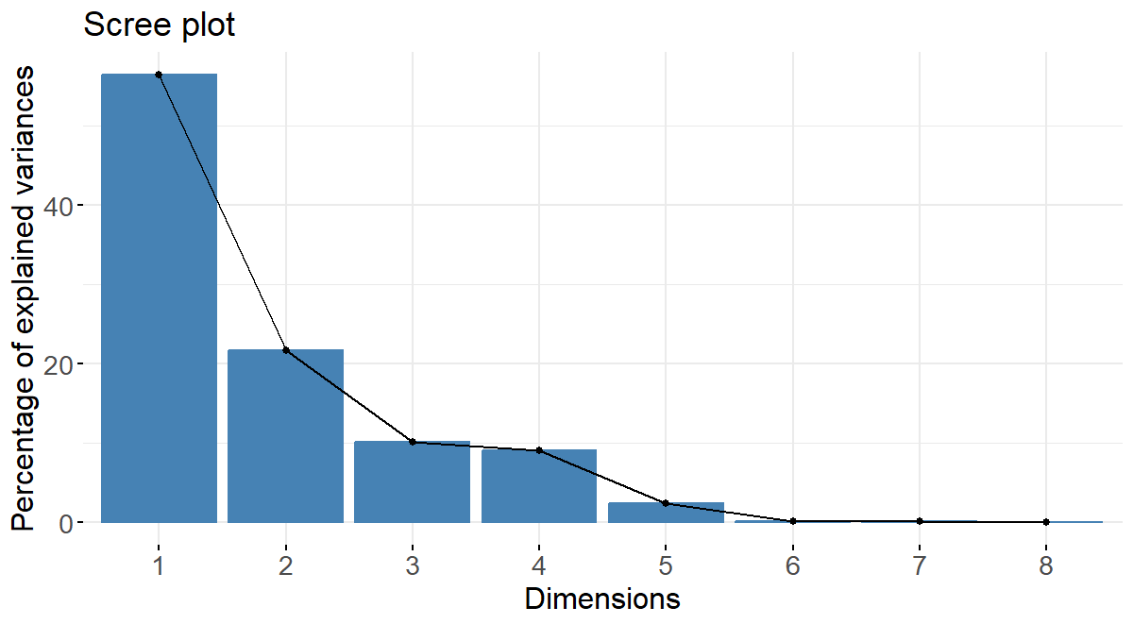


**Supplementary Fig. 2** Scree plot of PCA showing the percentage of explained variance by each dimension.


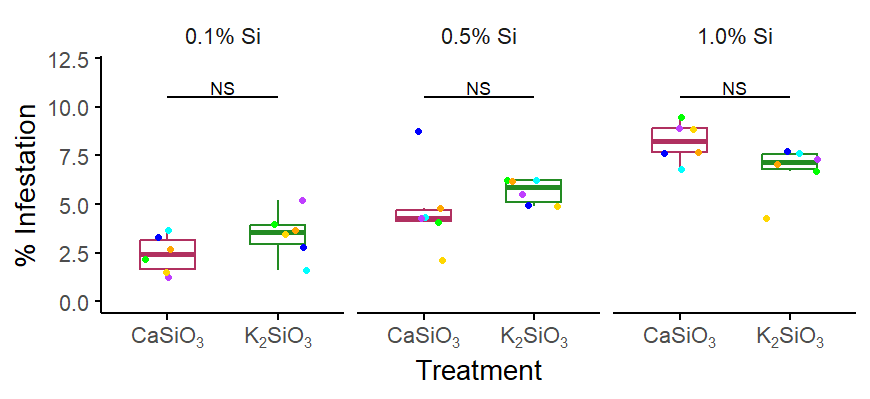


**Supplementary Fig. 3** Comparison of percent leaf infestation after the third spray of CaSiO₃ and K₂SiO₃ at silicon concentrations of 0.1%, 0.5% and 1.0%. Results showed no statistically significant differences between treatments (0.1% Si, t_10_ = -1.62, p = 0.1357; 0.5% Si, t_10_ = -1.03, p = 0.3422; 1.0% Si, t_10_ = 2.17, p = 0.0571). Data were analyzed using an individual sample t-test at p < 0.05. Here, NS = non-significant.
